# Lack of Association of CD55 Receptor Genetic Variants and Severe Malaria in Ghanaian Children

**DOI:** 10.1534/g3.116.036475

**Published:** 2017-01-18

**Authors:** Kathrin Schuldt, Christa Ehmen, Juergen Sievertsen, Jennifer Evans, Juergen May, Daniel Ansong, Birgit Muntau, Gerd Ruge, Christian Timmann, Tsiri Agbenyega, Rolf D. Horstmann, Thorsten Thye

**Affiliations:** * Department of Molecular Medicine, Bernhard Nocht Institute for Tropical Medicine, 20359 Hamburg, Germany; † Infectious Disease Epidemiology Group, Bernhard Nocht Institute for Tropical Medicine, 20359 Hamburg, Germany; ‡ School of Medical Sciences, Kwame Nkrumah University of Science and Technology, Kumasi, Ghana

**Keywords:** malaria, CD55, DAF, high-resolution screen, genetic association study

## Abstract

In a recent report, the cellular receptor CD55 was identified as a molecule essential for the invasion of human erythrocytes by *Plasmodium falciparum*, the causal agent of the most severe form of malaria. As this invasion process represents a critical step during infection with the parasite, it was hypothesized that genetic variants in the gene could affect severe malaria (SM) susceptibility. We performed high-resolution variant discovery of rare and common genetic variants in the human *CD55* gene. Association testing of these variants in over 1700 SM cases and unaffected control individuals from the malaria-endemic Ashanti Region in Ghana, West Africa, were performed on the basis of single variants, combined rare variant analyses, and reconstructed haplotypes. A total of 26 genetic variants were detected in coding and regulatory regions of *CD55*. Five variants were previously unknown. None of the single variants, rare variants, or haplotypes showed evidence for association with SM or *P. falciparum* density. Here, we present the first comprehensive analysis of variation in the *CD55* gene in the context of SM and show that genetic variants present in a Ghanaian study group appear not to influence susceptibility to the disease.

Globally, malaria killed ∼438,000 humans in the year 2015 (World Health Organization 2015). The pathogen causing SM is *Plasmodium falciparum*, a protozoan parasite, which is transmitted to the human host by the mosquito vector *Anopheles* spec. Humans get infected during a blood meal of the female mosquito, when parasites are injected into the skin together with the insect’s saliva.

The clinical picture of SM is complex and includes a number of life-threatening signs, such as severe malaria anemia (SMA), cerebral malaria (CM), hyperparasitemia, respiratory distress, and prostration. Besides parasitic and environmental determinants, several host factors are known to influence the risk of developing SM and its associated complications. These factors include age and immune state, as well as the genetic make-up of an infected individual. A study by Mackinnon *et al.* (2005) estimated that 25% of the variation in SM susceptibility could be attributed to the genetic background of the human host. Over the past decades, many candidate and a few genome-wide association studies on SM have repeatedly shown the impact of human genetic variation on disease susceptibility (Kwiatkowski 2005; Jallow *et al.* 2009; Timmann *et al.* 2012; Band *et al.* 2013).

CD55, a surface molecule on human red blood cells (RBCs), was recently identified as an essential receptor for the invasion of human RBCs by malaria parasites (Egan *et al.* 2015). CD55, also known as decay accelerating factor for complement (DAF), is a glycosylphosphatidylinositol (GPI)-anchored protein that is broadly distributed among hematopoietic and nonhematopoietic cells. Its main function is the regulation of the complement cascade on the cell surface (Lublin and Atkinson 1989). In addition, it carries the antigen determinants for the Cromer blood group system (CROM). In a forward genetic screen, Egan *et al.* (2015) showed that CD55-knockdown cells were resistant to invasion by laboratory and by clinical *P*. *falciparum* isolates, indicating that CD55 is a critical host factor for all *P. falciparum* strains. Results from invasion assays also revealed that CD55 is not required for the initial attachment of merozoites to the RBCs, but plays an essential role later in the process, possibly during tight junction formation. Regarding naturally occurring genetic variation in *CD55*, data from the 1000 Genomes Project (www.1000Genomes.org) revealed an enrichment of two structural CD55 polymorphisms, R52L (CROM3) and A227P (CROM1), in individuals with ancestral exposure to malaria (Egan *et al.* 2015).

Taken together, there is substantial evidence that human genetic variation affecting CD55 integrity on the surface of RBCs could influence SM phenotypes, rendering *CD55* a promising candidate for association studies. Moreover, CD55 has been discussed as a potential drug target (Tham and Kennedy 2015) and, as such, information on the degree of sequence conservation among malaria-exposed populations is crucial.

Here, we screened a large case-control study group comprising more than 1700 subjects (May *et al.* 2007) from the Ashanti Region in Ghana, West Africa, for genetic variation in the *CD55* gene and assessed the influence of common and rare coding variants, and the impact of *CD55* haplotypes on SM phenotypes.

## Materials and Methods

### SM case-control group

The SM case-control group comprised 831 SM cases and 903 control individuals from the Ashanti Region in Ghana. SM was defined according to World Health Organization (WHO) criteria as described (May *et al.* 2007), and control subjects were unaffected healthy individuals. Within the SM case group, 507 (61.0%) presented with SMA and 165 (19.9%) had CM (Table 1). The majority of the SM cases were also affected by additional severe manifestations such as prostration, hyperparasitemia, or hyperlactatemia, which were partly overlapping. Parasite density was determined by counting *P. falciparum* asexual blood stages in Giemsa-stained blood smears (May *et al.* 2007). The median age of cases and control individuals was 18 and 27 months, respectively (ranges 3–147 months in the case group and 4–161 months in the control group). Phenotyping, recruitment of study subjects, and DNA extraction methods have previously been described in detail (May *et al.* 2007; Schuldt *et al.* 2011). After genotyping and evaluation of association results for variant rs7542430, additional DNA samples from 2704 study subjects were used for genotyping in order to confirm initial findings. These subjects were cases and controls that were part of the same original study (May *et al.* 2007).

**Table 1:** Characteristics of severe malaria case-control study

		Median Age (Range in Months)	Ethnic Group (%)		
	*n*		Akan	Northerner	Ewe
Case-control study					
Severe malaria	831	18 (3–147)	68.3	29.5	2.2
Controls	903	27 (4–161)	84.1	14.2	1.7
Cases stratified by major phenotypes					
Severe anemia*^a^*	506	13 (3–132)	69.7	28.2	2.1
Cerebral malaria*^b^*	166	24 (3–147)	66.9	30.7	2.4

a Severe malaria anemia (hemoglobin level < 5 g/dl) with or without additional complications including cerebral malaria.

b Cerebral malaria (Blantyre Coma Score [BCS] < 3) with or without additional complications including severe anemia.

### Variant discovery and genotyping

The gene encoding CD55 is located on chromosome 1 and spans ∼40 kb of genomic DNA. In order to screen the *CD55* locus (chr1:207,494,817–207,534,311, hg19) for genetic variants, DNA from 1734 unrelated Ghanaian individuals (831 SM cases and 903 controls) was used for high resolution melting analyses (HRM). Therefore, DNA samples were amplified by PCR using primers that capture the 11 *CD55* exons, including a small proportion of their flanking regions as well as 140 bp of untranslated sequence upstream of the transcription start site. Oligonucleotides were designed using LightCycler Probe Design Software 2.0 (Roche Applied Science) against reference transcript NCBI NM_000574.4. Sequences of oligonucleotides and PCR conditions for HRM assays are listed in Supplemental Material, Table S1. Previously unknown single nucleotide polymorphisms (SNPs) and singletons were confirmed by resequencing of genomic DNA. Information about all novel variants detected in our study group was submitted to NCBI dbSNP and will be released in Build 148. In addition, two promoter variants, rs28371586 and rs7542430, were genotyped by allele-specific hybridization in a Roche Lightcycler device (Table S2). Consequences of discovered amino acid missense mutations were analyzed by Polyphen2 and SIFT predictions (Ng and Henikoff 2001; Adzhubei *et al.* 2010).

### Calculation of linkage disequilibrium (LD)

Haploview was used to generate the LD plot of the *CD55* genomic region (Barrett *et al.* 2005). The LD calculation was based on healthy control individuals and included all variants with a global minor allele frequency (MAF) ≥ 1%.

### Association analyses of genetic variants with MAF ≥ 1%

Tests for association of seven variants with global MAFs ≥ 1% were done by logistic and linear regression analyses in PLINK v1.9 assuming an additive mode of inheritance (Purcell *et al.* 2007). In the regression models, ethnic group, age, and gender were used as covariates. The logarithm of the parasite density was used as a quantitative phenotype in a linear regression analysis in PLINK. In order to account for multiple testing, a correction factor of seven was used; hence, a p-value < 0.007 (0.05/7) was considered significant. A factor of seven was applied because the correlation between the seven SNPs was negligible (all pairwise *r*^2^-values < 0.05 Figure S1), hence the associations tested represented seven independent tests. All variants were tested for fulfilling the Hardy–Weinberg equilibrium (HWE) in PLINK and were found to comply with frequencies under HWE.

Power for detecting effects of variants in the case-control study was estimated with CATS (Skol *et al.* 2006). For power estimations, a disease prevalence of 2% for SM and p < 0.007 were assumed. Power calculations for single association tests resulted in a power of 99, 95, and 53% to detect a genotype–phenotype association in the SM, SMA, and CM study groups, respectively, when assuming a genotype relative risk of at least two and a MAF ≥ 5%.

### Haplotype-based association testing

Full-length haplotype analyses were done for the case-control group with the HaploStats package v1.4.4 (Schaid *et al.* 2002) in R (version 3.1.0; http://www.r-project.org), including reconstructed haplotypes with estimated global frequencies ≥ 1%. In addition, subhaplotypes were evaluated in sliding window analyses capturing a minimum of two and a maximum of six alleles. SM, as well as the major manifestations SMA and CM, and parasite density (logarithmic scale) were used as phenotypes, and the three covariates ethnic group, age, and gender were included in the model. Empirical p-values were computed using default values.

### Association analyses of rare variants

Four different methods integrated in variant tools were used to assess the overall genetic burden due to rare variants (global MAF < 1%) (San Lucas *et al.* 2012). These included: (i) the combined and multivariate collapsing (CMC) method described by Li and Leal (2008), (ii) the weighted sum statistic (WSS) described by Madsen and Browning (2009), (iii) the variable-threshold (VT) model described by Price *et al.* (2010), and (iv) the C(α) test described by Neale *et al.* (2011). Variants were pooled on the basis of their MAF and their cumulative effect was tested in univariate and multivariate analyses, in which age, gender, and ethnic group were included as covariates. Tests for association of rare variant carrier status in *CD55* were done for SM, SMA, CM, or hyperparasitemia, defined as > 200,000 asexual parasites/μl blood.

### Ethics statement

Ethical clearance was granted by the Committee for Research, Publications and Ethics of the School of Medical Sciences, Kwame Nkrumah University of Science and Technology, Kumasi, Ghana. All procedures were explained to parents or guardians of the participating children in the local language, and written or thumb-printed informed consent was obtained.

### Data availability

The CD55 mRNA reference sequence for design of HRM assays is accessible under NCBI NM_000574.4. Oligonucleotides and conditions for HRM reactions and for SNP genotyping are found in Table S1 and Table S2. Genomic locations and frequencies of the SNPs present in this Ghanaian study group are shown in Table 2. Information on previously unknown genetic variants was submitted to NCBI dbSNP and assigned SNP IDs are included in Table 2.

**Table 2:** Genetic variants in the *CD55* gene detected in 1734 Ghanaian children

	SNP ID	Position Chr1 (Build 37)	Relative Position*^a^*	Amino Acid Exchange	Novel	MAF Cases (*N* = 831)	MAF Controls (*N* = 903)	Polyphen2 Prediction*^b^*	SIFT Prediction*^c^*
1	rs6685886	207,494,218	−893C > A			0.27	0.23		
2	rs28371586	207,494,913	−198C > G			0.02	0.03		
3	rs879066510	207,495,089	−21C > G		Yes	-	0.0006		
4	rs7542430	207,495,116	c.6C > G	T2T		0.01	0.02		
5	rs28371587	207,495,224	c.100 + 14C > T			0.002	0.002		
6	rs28371588	207,495,781	c.155G > T	R52H		0.04	0.04	Benign	Tolerated
7	rs147474393	207,495,871	c.245T > G	L82R		0.008	0.01	Benign	Tolerated
8	rs28371603	207,497,911	c.294C > T	C98C		0.005	0.007		
9	rs2564979	207,498,939	c.479-28T > A			0.03	0.04		
10	rs150286783	207,500,169	c.651G > A	L217L		-	0.0005		Tolerated
11	rs878953964	207,504,465	c.677C > T	P226L	Yes	—	Singleton	Probably damaging	Tolerated
12	rs60822373	207,504,467	c.679G > C	A227P		—	Singleton	Probably damaging	Tolerated
13	rs566298946	207,504,537	c.749C > T	T250M		—	Singleton	Probably damaging	Damaging
14	rs756597715	207,510,066	c.882A > G	P294P		—	Singleton		
15	rs779822800	207,510,067	c.883A > G	T295A		Singleton	—	Possibly damaging	Tolerated
16	rs879028717	207,510,084	c.900	T300T	Yes	—	Singleton		
17	rs774684822	207,510,665	c.980-9A > G			Singleton	—		
18	rs116473488	207,510,691	c.997G > A	V333I		0.02	0.02	Benign	Tolerated
19	rs550337244	207,510,708	c.1014G > A	K338K		0.001	0.002		
20	rs150172588	207,510,725	c.1031C > T	T344I		0.003	0.003	Possibly damaging	Tolerated
21	rs28371628	207,512,734	c.1081-8G > A			0.08	0.07		
22	rs542054808	207,513,741	c.1081 + 979C > T			Singleton	—		
23	rs879071049	207,513,772	c.1081 + 1010G > A		Yes	Singleton	—		
24	rs138016651	207,513,861	c.1081 + 1099C > T			0.005	0.007		
25	rs375489214	207,532,908	c.1099T > C	V406A		—	Singleton	n.a.	Tolerated
26	rs878906671	207,532,925	c.1117G > T	D412Y	YES	Singleton	—	n.a.	Damaging

SNP ID, single nucleotide polymorphism identifier; Chr1, chromosome 1; MAF, minor allele frequency; n.a., not applicable.

a Reference sequence NCBI NM_000574.4.

b URL: http://genetics.bwh.harvard.edu/pph2.

c URL: http://sift.jcvi.org/www/SIFT_chr_coords_submit.html.

## Results

Variant discovery of *CD55* was conducted in 1734 unrelated individuals from the Ashanti Region in Ghana. The study group comprised 831 SM cases and 903 apparently healthy control individuals. The genomic region screened included coding and regulatory regions of *CD55* as well as the intron–exon boundaries. As a result, a total of 26 SNPs were found to be present in the study group. Of these, five variants were previously unknown (Table 2).

Nineteen variants were found to be very rare with MAFs below 1% including 11 singletons. Only three SNPs with MAFs ≥ 1% were found that caused nonsynonymous amino acid exchanges in the receptor protein. These were R52H, L82R, and V333I, with MAFs of 4, 1, and 2% in the control group, respectively. According to Polyphen2 and SIFT predictions all three substitutions were classified as benign. With the exception of V406A, all other missense mutations, all being singletons, were predicted to be probably damaging or damaging (Table 2). Seven variants were found to be more frequent than 1%, and these were initially used in single variant association tests with SM, SMA, CM, or parasite density. The findings are summarized in Table 3.

**Table 3:** Association tests of *CD55* SNPs (MAF > 1%) with severe malaria phenotypes

			
				Severe Malaria (*N*_cases_ = 831, *N*_controls_ = 903)		Severe Malaria Anemia (*N*_cases_ = 507, *N*_controls_ = 903)		Cerebral Malaria (*N*_cases_ = 165, *N*_controls_ = 903)		Parasite Density (Log) (*N*_cases_ = 767)	
No.	SNP ID	Maj/Min Allele	MAF (Aff/Unaff)	OR (95% C.I.)	p-Value	OR (95% C.I.)	p-Value	OR (95% C.I.)	p-Value	β (SE)	p-Value
1	rs6685886	C/A	0.27/0.23	1.12 (0.95–1.31)	0.181	1.15 (0.95–1.39)	0.144	1.01 (0.83–1.44)	0.525	−0.09 (0.12)	0.476
2	rs28371586	C/G	0.02/0.03	0.84 (0.54–1.32)	0.457	0.73 (0.41–1.20)	0.229	1.21 (0.61–2.40)	0.587	0.54 (0.37)	0.147
3	rs7542430	C/G	0.01/0.02	0.44 (0.25–0.79)	0.006	0.40 (0.20–0.81)	0.011	0.84 (0.36–1.97)	0.681	−0.69 (0.48)	0.158
4	rs28371588	G/T	0.04/0.04	0.88 (0.61–1.25)	0.469	0.83 (0.54–1.28)	0.393	1.07 (0.59–1.94)	0.829	0.03 (0.30)	0.910
5	rs2564979	T/A	0.03/0.04	0.82 (0.55–1.21)	0.312	0.85 (0.55–1.33)	0.488	0.57 (0.25–1.29)	0.176	0.11 (0.32)	0.740
6	rs116473488	G/A	0.02/0.02	0.86 (0.52–1.41)	0.556	0.86 (0.48–1.53)	0.599	0.56 (0.20–1.60)	0.278	0.02 (0.42)	0.967
7	rs28371628	G/A	0.08/0.07	1.24 (0.93–1.64)	0.141	1.14 (0.82–1.59)	0.436	1.33 (0.84–2.01)	0.228	−0.04 (0.22)	0.849

Severe malaria, severe malaria anemia, cerebral malaria, and parasite density results of logistic regression analyses (linear regression for parasite density) are presented, assuming an additive mode of inheritance adjusted for gender, age, and ethnicity. No., number; SNP ID, single nucleotide polymorphism identifier; Maj, major; Min, minor; MAF, minor allele frequency; Aff, affected individuals; Unaff, unaffected individuals; OR, odds ratio.

As a result, the G allele of rs7542430 was found to be associated with a reduction in risk of SM (odds ratio (OR) 0.44, 95% C.I. 0.25–0.79, and p = 0.006). However, when additional subjects of the study were analyzed (*n*_cases_ = 2588 and *n*_controls_ =1850) the association did not hold (OR 0.92, 95% C.I. 0.67–1.26, and p = 0.598).

Reconstruction of haplotypes generated eight full-length haplotypes with a global MAF above 1% (Table 4). Of these, haplotype CD55-1 was the most prominent one with an estimated frequency in the control subjects of 57%. Haplotype CD55-2 was found more frequently in SM cases than in controls (AF 25% in cases and 21% in controls). This difference was statistically significant in the haplotypic-specific score test (p = 0.03, p_empirical_ = 0.04). A similar result was found for haplotype CD55-8, which had an estimated AF of 1% in the SM case group and 2% in the control group, and haplotype-specific p-values of 0.01 (Table 4). However, after correction for multiple hypotheses testing, the global test-statistic produced p-values of 0.12 and 0.32, indicating no association of full-length haplotypes with any of the phenotypes.

**Table 4:** Association tests of *CD55* haplotypes of ≥ 1% frequencies with severe malaria

		
			Severe Malaria *N*_cases_ = 831, *N*_controls_ = 903		Severe Malaria Anemia *N*_cases_ = 522, *N*_controls_ = 903		Cerebral Malaria *N*_cases_ = 203, *N*_controls_ = 903		Parasite Density (Log) *N*_cases_ = 767	
Haplotype	1-2-3-4-5-6-7*^a^*	Frequency (Aff/Unaff)	p-Value*^b^*	Simulated p-Value*^c^*	p-Value*^b^*	Simulated p-Value*^c^*	p-Value*^b^*	Simulated p-Value*^c^*	p-Value*^b^*	Simulated p-Value*^c^*
CD55-1	CCCGTGG	0.55/0.57	0.475	0.487	0.673	0.665	0.325	0.342	0.428	0.423
CD55-2	ACCGTGG	0.25/0.21	0.031	0.036	0.027	0.024	0.100	0.108	0.298	0.265
CD55-3	CCCGTGA	0.08/0.07	0.133	0.116	0.509	0.507	0.112	0.111	0.820	0.821
CD55-4	CCCTTGG	0.04/0.04	0.403	0.414	0.361	0.347	0.951	0.941	0.689	0.729
CD55-5	CCCGAGG	0.03/0.03	0.255	0.265	0.368	0.346	0.087	0.084	0.796	0.802
CD55-6	CGCGTGG	0.02/0.03	0.484	0.489	0.372	0.375	0.969	0.968	0.629	0.630
CD55-7	CCCGTAG	0.02/0.02	0.524	0.534	0.630	0.639	0.232	0.255	0.894	0.889
CD55-8	ACGGTGG	0.01/0.02	0.011	0.007	0.024	0.031	0.659	0.646	0.124	0.120

Aff, affected individuals; Unaff, unaffected individuals; SNP, single nucleotide polymorphism.

a Refers to SNP number as designated in Table 2.

b Results of haplotypic-specific score tests adjusted for gender, age, and ethnicity assuming an additive mode of inheritance.

c Simulation p-values are computed based on a permuted reordering of the trait and covariates in HaploStats (Schaid *et al.* 2002).

In order to test for an accumulation of rare variants in either the case or control group, four different algorithms were applied: the CMC method, the WSS, the VT model, and the C(α) test. None of the approaches, which included univariate and multivariate collapsing tests, provided evidence for a joint effect of rare variants on the phenotypes tested (Table 5).

**Table 5:** *CD55* rare variant analyses including eight SNPs with MAF ≤ 1%


	Severe Malaria	Severe Malaria Anemia	Cerebral Malaria	Hyperparasitemia (> 200,000/µl)
	*N*_cases_ = 831, *N*_controls_ = 903	*N*_cases_ = 507, *N*_controls_ = 903	*N*_cases_ = 165, *N*_controls_ = 903	*N*_cases_ = 220, *N*_controls_ = 547
	p-Value	p-Value	p-Value	p-Value
Univariate tests			
CMC	0.952	0.979	0.890	0.507
WSS	0.954	0.976	0.835	0.451
C(α)	0.400	0.412	0.777	0.641
Multivariate tests*^a^*				
CMC	0.935	0.963	0.904	0.423
WSS	0.972	0.970	0.907	0.491
VT test (13 variants)	0.966	0.982	0.829	0.896

CMC, combined and multivariate collapsing; WSS, weighted sum statistic; VT, variable-thresholds methods.

a Adjusted for age, gender, and ethnic group.

## Discussion

Following a recent report on CD55 and its essential role for *P. falciparum* survival, it was hypothesized that host genetic variation of CD55 may have an impact on SM disease severity. Here, we analyzed variants in the coding sequence and regulatory regions of *CD55* in a large SM case-control study from Ghana. The findings of our study do not support an effect of genetic variants tested on parasite density or susceptibility to major clinical manifestations of SM. Only 3 amino acid-changing variants were detected that had MAFs between 1 and 4%, indicating a relatively high degree of sequence conservation in the *CD55* exonic sequence in the Ghanaian population.

To our knowledge, this is the first study to identify novel genetic variants of *CD55* in African subjects and test thoroughly for association with SM phenotypes. In their report, Egan *et al.* (2015) described two nonsynonymous *CD55* polymorphisms that were significantly enriched in populations with ancestral or current exposure to malaria: CROM3 (R52L) in African Americans and in the Yoruba ethnic group from Nigeria, and CROM1 (A227P) only present in the Yoruba. The CROM3 genotype reached frequencies of 4.9% in African Americans and 5.7% in the Yoruba, but was not present in our Ghanaian study group. Instead, we found the coding variant R52H, which had a MAF of 4% in the case and control groups and did not show evidence for an association with any of the SM phenotypes. CROM1 was found once in the control group, indicating a very low frequency in the Ghanaian population. If this variant influenced disease outcome at all, it would have a very low impact on disease risk at population level. Because Egan *et al.* (2015) classified populations of the 1000 Genomes Project into high and low malaria-exposed populations and performed a simple comparison of MAFs between these two groups, it is possible that this analysis produced spurious results, as it may solely capture interethnic differences instead of malaria-specific associations. Moreover, the diversity of allele frequencies across the analyzed 1000 genomes populations may be driven by other pathogens that exert selective pressure on the human genome. According to Egan *et al.* (2015), CD55 not only functions as a receptor for *P. falciparum* merozoites, but also for viral and bacterial pathogens. Therefore, it could also be possible to envisage a selective pressure by pathogens other than *P. falciparum*, resulting in different frequencies of *CD55* genetic variants.

Of note, a lack of association in the Ghanaian study group with the analyzed *CD55* variants does not exclude significant associations with the same variants in other populations exposed to malaria. There are several coevolutionary effects between *P. falciparum* and its human host that have shaped the human genome (Kwiatkowski 2005; Bongfen *et al.* 2009). As a consequence, some types of adapted mechanisms based on structural genetic variation may be restricted to particular human subpopulations, as has been described for the protective HbC variant, which is found at high frequencies in West African populations but is not present in other malaria-exposed populations (Taylor *et al.* 2013). Likewise, there may be true associations between genetic variants and SM in additional chromosomal regions of the human genome that influence CD55 expression. Here, we screened the *CD55* coding sequence, intron–exon boundaries, and part of the promoter region, because the main focus was laid on structural variation or splice variants of the CD55 receptor, which are likely to influence invasion of RBC by *P. falciparum* merozoites.

Another reason why existing genotype–phenotype associations may remain obscure is lack of power. The power of a study depends on the number of study participants and the MAFs and effect sizes of the alleles tested. For single SNP associations, our study provided enough power for all SNPs > 5% in the SM and SMA study groups. However, the study was underpowered when analyzing SNPs with lower frequencies in the single SNP association tests. Hence, low frequency variants were analyzed using several collapsing approaches that have been developed in order to increase the power for association studies of rare variants.

In addition, there may be true associations between genetic variants and SM in chromosomal regions of the human genome that influence CD55 expression, but that were not covered in this study. Here, we mainly screened the *CD55* coding sequence, including intron–exon boundaries and part of the promoter region, because the main focus was laid on structural variation or splice variants of the CD55 receptor, which are likely to influence invasion efficiency. In this regard, exact mechanisms of how CD55 expression is regulated and the genetic variation with potential influence on expression levels on the RBC surface remains to be explored. For instance, a study that evaluated expression quantitative trait loci (eQTLs) in blood cells in samples from over 5300 donors did not identify any variation that functions as eQTLs for CD55 (Westra *et al.* 2013). One reason may be that the study did not include individuals of African ancestry. In this respect, CD55 expression studies in RBCs originating from African individuals would be helpful to specifically determine eQTLs for CD55 in RBCs.

## Supplementary Material

Supplemental material is available online at www.g3journal.org/lookup/suppl/doi:10.1534/g3.116.036475/-/DC1.
