# Supplementary material for: Lack of Association of CD55 Receptor Genetic Variants and Severe Malaria in Ghanaian Children
Source: G3 (Bethesda). 2017 Jan 18;7(3):859–64. doi: 10.1534/g3.116.036475 (PMC5345716; doi:10.1534/g3.116.036475)
Supplement: Supplementary file 1 [file 859FigureS1.docx]

Figure S1 Linkage disequilibrium structure at *CD55* gene locus


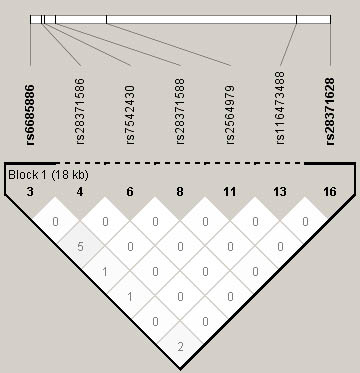


LD pattern is represented by pairwise r^2^ values between SNPs with MAF>1% based on genotypes of 831 severe malaria cases and 903 controls. r^2^ values (x100) for each comparison is given in the squares. White squares represent r^2^ values equal to 0. Squares that are shades of grey represent values between 0 and 1.
