# Supplementary material for: Lack of Association of CD55 Receptor Genetic Variants and Severe Malaria in Ghanaian Children
Source: G3 (Bethesda). 2017 Jan 18;7(3):859–64. doi: 10.1534/g3.116.036475 (PMC5345716; doi:10.1534/g3.116.036475)
Supplement: Supplementary file 2 [file 859TableS1.docx]

Table S1 Oligonucleotides and PCR conditions for *CD55* high resolution melting assays

|  | Gene region | Length of amplicon  in bp | Oligonucleotides | Annealing temperature | Additional or deviant reagents in 10 μl reaction |
| --- | --- | --- | --- | --- | --- |
| 1 | Exon 1 | 288 | \| CD55-Ex1-2-F2 CGCCCAAAGCACTCATTTAACTGG  CD55-Ex1-2-R GACTTGGACCTCCCACCCAG \| \| --- \| | HotStart touchdown, 11 cycl.65-55°C,40 cycl,58 °C | 1,5 mM MgCl_2,_ 5% DMSO  0.5 µM Forward,0.5 µM Reverse |
| 2 | Exon 2 | 259 | CD55-Ex2-F CTTCAGTTCTGCTTTTGTCTCCCTAG  CD55-Ex2-R AGATACATTCCATTCCCAGAACTTTTC | 58°C | 2,5 mM MgCl_2_  1x Solis Solution S |
| 3 | Exon 3 | 313 | CD55-Ex3-F ATTGATACTACATTTTTTGTTGCTGCT  CD55-Ex3-R CTAGCATGAATGAAGGAAGGGATAC | HotStart  53°C | 2,5 mM MgCl_2,_ 5% DMSO  1x Solis Solution S |
| 4 | Exon 4 | 236 | CD55-Ex4-F ACATATCTACCACCTCACATAGTTAC  CD55-Ex4-R ATTTGTTTTCTCATTTCCTAAATACAGGG | 61°C | 2,5 mM MgCl_2_  1x Solis Solution S |
| 5 | Exon 5 | 203 | CD55-Ex5-F ACTCTTTTTTCTTCCCCTGTTGCT  CD55-Ex5-R2 ACTGTGCTAATATTCTTAAGGGGC | HotStart-Touchdown  11 cycl.65-55°C,40 cycl.  58 °C | 1,5 mM MgCl_2,_ 5% DMSO  0.5 µM Forward, 0.5 µM Reverse  1x Solis Solution S |
| 6 | Exon 6 | 305 | CD55-Ex6-F ACTAGTTTTATTTATTTAAAAGATGTTGG  CD55-Ex6-R TTGAATGTCTGCAACCCAC | HotStart  53°C | 2,5 mM MgCl_2,_ 5% DMSO  1x Solis Solution S |
| 7 | Exon 7 | 221 | CD55-Ex7-F AATGTTAATGTGGCCAGCAATATTTAG  CD55-Ex7-R GTGGTGTGTTTTTGAAGAACTGATG | 61°C | 2,5 mM MgCl_2_  1x Solis Solution S |
| 8 | Exon 8 | 164 | CD55-Ex8-F CAAGAGTACACAAAKATTCCCTTCTG  CD55-Ex8-R GCACATTAGATCCATACTCAGTAAACC | HotStart  53°C | 2,5 mM MgCl_2,_ 5% DMSO  1x Solis Solution S |
| 9 | Exon 9 | 157 | CD55-Ex9-F TAGCAAATCAATGACTTTAACAAATTTTTG  CD55-Ex9-R TATCTCCCAGGAATATGGATTGTATATC | 58°C | 2,5 mM MgCl_2,_ 1x Solis Solution S |
| 10 | Exon 10 | 234 | CD55-Ex10-F ATATGCTAGATTTGTTTTCTTTCTTTTC  CD55-Ex10-R TCCTGTCTCTACAAAAAATAAATTTCAA | 58°C | 2 mM MgCl_2,_ 1x Solis Solution S |
| 11 | Exon 11 | 324 | CD55-Ex11-1-F GCACCCCAAATTAACTGATTCTT  CD55-Ex11-1-R ACACATCTTAAAGACAATGAAAGCAT | 58°C | 2 mM MgCl_2,_ 1x Solis Solution S |

Unless otherwise specified the following reagents are used in all reactions (PCR components: Solis Biodyne): 1x PCR-Buffer, 200 µM dNTPs, 1 unit Taq, 0,3 µM of each primer (Biomers), 1x EvaGreen, 20 ng DNA. Cycle protocol: 3 min initial denaturation 94°C (for HotStart reactions 15 min), 45 cycl. 30 sec denaturation 94°C, 1 min annealing as mentioned below, 1 min 72°C (for HotStart reactions 68°C), 5 min final extension 72°C (for HotStart reactions 68°C). Cycle protocol for Hotstart touchdown reactions: 15 min initial denaturation 94°C ; 11 cycles 20 sec denaturation 94°C, 30 sec touchdown 65°- 55°C in 1°C steps per cycle, 30 sec extension at 68°C, additional 40 cycles with 20 sec denaturation 94°C, 30 sec annealing at 58°C , 30 sec extension at 68°C; 5 min final extension 68°C.
