# Supplementary material for: Lack of Association of CD55 Receptor Genetic Variants and Severe Malaria in Ghanaian Children
Source: G3 (Bethesda). 2017 Jan 18;7(3):859–64. doi: 10.1534/g3.116.036475 (PMC5345716; doi:10.1534/g3.116.036475)
Supplement: Supplementary file 3 [file 859TableS2.docx]

Table S2 Oligonucleotides and PCR conditions for *CD55* SNP genotyping

|  | rs_ID | Oligonucleotides | Annealing temperature | Additional or deviant reagents in 10 μl reaction |
| --- | --- | --- | --- | --- |
| 1 | rs6685886 | \| rs6685886-F aggctatttattctaaagggtttgtaac \| \| --- \| \| rs6685886-R tttacgaaagacaccactttgggattac \| \| rs6685886-A gtttgtttttattgttatcccacccc-6FAM \| \| rs6685886-S BMN5-caccgccccgag-Spacer C3 \| | 60°C | 2 mM MgCl_2,_ 5 µg BSA  1x Solis Solution S |
| 2 | rs28371586 | \| rs28371586-F \| TGTTTACGTAGCGAGGAGATATTTAGG \| \| --- \| --- \| \| rs28371586-R \| GCAATACCAGTTAAATGAGTGCTTTGG \| \| rs28371586-A \| Cy5-ACCCGTCTTGTTTGTCCC-Spacer-C3 \| \| rs28371586-S \| ACAACAAACCCCTACTC-Fluorescein \| | 52°C | 2.0 mM MgCl_2,_ 5 µg BSA  1x Solis Solution S |
| 3 | rs7542439 | \| rs7542430-F \| CCCAAAGCACTCATTTAACTGGTATTG \| \| --- \| --- \| \| rs7542430-R \| CTCCCTTTCGATTTTGCCAGGCTTG \| \| rs7542430-A \| CTTGTTCTAACCCGGCGCG-Fluorescein \| \| rs7542430-S \| Cy5-CATGACCGTCGCG-Spacer-C3 \| | 58°C | 2.0 mM MgCl_2,_ 5 µg BSA  1x Solis Solution S |
